# Coadministration of FTY720 and rt-PA in an experimental model of large hemispheric stroke–no influence on functional outcome and blood–brain barrier disruption

**DOI:** 10.1186/2040-7378-5-11

**Published:** 2013-10-28

**Authors:** Aijia Cai, Frieder Schlunk, Ferdinand Bohmann, Sepide Kashefiolasl, Robert Brunkhorst, Christian Foerch, Waltraud Pfeilschifter

**Affiliations:** 1 Department of Neurology, University Hospital, Goethe University Frankfurt, Frankfurt, Germany; 2 Department of General Pharmacology and Toxicology, University Hospital, Goethe University Frankfurt, Frankfurt, Germany; 3 Department of Neurosurgery, University Hospital, Goethe University Frankfurt, Frankfurt, Germany

**Keywords:** Stroke, MCAO, Thrombolysis, FTY720, Fingolimod, Sphingolipid, S1P1 receptor, Brain, Hemorrhage, Blood–brain barrier

## Abstract

**Background:**

Systemic thrombolysis with recombinant tissue plasminogen activator (rt-PA) is the standard of acute stroke care. Its potential to increase the risk of secondary intracerebral hemorrhage, especially if administered late, has been ascribed to its proteolytic activity that has detrimental effects on blood–brain barrier (BBB) integrity after stroke. FTY720 has been shown to protect endothelial barriers in several disease models such as endotoxin-induced pulmonary edema and therefore is a promising candidate to counteract the deleterious effects of rt-PA. Besides that, every putative neuroprotectant that will be eventually forwarded into clinical trials should be tested in conjunction with rt-PA.

**Methods:**

We subjected C57Bl/6 mice to 3 h filament-induced tMCAO and postoperatively randomized them into four groups (n = 18/group) who received the following treatments directly prior to reperfusion: 1) vehicle-treatment, 2) FTY720 1 mg/kg i.p., 3) rt-PA 10 mg/kg i.v. or 4) FTY720 and rt-PA as a combination therapy. We measured functional neurological outcome, BBB disruption by quantification of EB extravasation and MMP-9 activity by gelatin zymography.

**Results:**

We observed a noticeable increase in mortality in the rt-PA/FTY720 cotreatment group (61%) as compared to the vehicle (33%), the FTY720 (39%) and the rt-PA group (44%). Overall, functional neurological outcome did not differ significantly between groups and FTY720 had no effect on rt-PA- and stroke-induced BBB disruption and MMP-9 activation.

**Conclusions:**

Our data show that FTY720 does not improve functional outcome and BBB integrity in large hemispheric infarctions, neither alone nor in conjunction with rt-PA. These findings stand in contrast to a recently published study that showed beneficial effects of FTY720 in combination with thrombolysis in a thrombotic model of MCAO leading to circumscript cortical infarctions. They might therefore represent a caveat that the coadministration of these two drugs might lead to excess mortality in the setting of a severe stroke.

## Background

Thrombolysis with recombinant human tissue plasminogen activator (rt-PA) is the only approved and evidence-based medical therapy for ischemic stroke. Within the narrow therapeutic time window of 4.5 h, it confers a clear net benefit to all stroke patients who receive thrombolysis which comes at the cost of an increased risk of secondary intracerebral hemorrhage for the individual patient, mostly caused by hemorrhagic transformation (HT) of the infarcted brain tissue [1].

Magnetic resonance imaging studies in acute stroke patients have shown that blood–brain barrier (BBB) disruption is significantly more prevalent in stroke patients who received thrombolysis than in untreated stroke patients [2]. Based on the description of HARM (hyperintense acute reperfusion marker) which was defined as the presence of gadolinium extravasation into the cerebrospinal fluid (CSF) space adjacent to the infarction on the fluid-attenuated inversion recovery (FLAIR) sequence of a follow up scan after gadolinium injection for a scan that took place a few hours earlier, it became evident that preceeding BBB disruption was present in 73% of patients who developed hemorrhagic transformation within the next hours [3].

These observations were supported by animal studies showing that cerebral ischemia leads to an increase of matrix-metalloproteinase (MMP) activity, especially of MMP-9 which follows the same time course as BBB disruption after experimental stroke and that both are aggravated by treatment with rt-PA [4] and correlate with HT [5]. Therefore, combination therapies with drugs that protect endothelial barrier function seem to be a reasonable approach to limit the risks of rt-PA treatment.

One of these substances is the sphingosine 1-phosphate analog FTY720, an immunomodulator that has been marketed in 2010 for the treatment of relapsing-remitting multiple sclerosis (MS). The presumed mechanism of this drug is the induction of peripheral lymphocytopenia that is caused by agonist-induced internalization of the S1P_1_ receptor of B and T lymphocytes which limits lymphocyte egress from primary lymphoid organs [6]. But beside its effects on immune cell trafficking, FTY720 also has effects on neurons, glia, and progenitor cells in the brain and an effect on the BBB can be presumed [7]. Interestingly, FTY720 treatment led to a downregulation of inflammatory genes including MMP-9 and to an increased expression of its counterpart tissue inhibitor of metalloproteinase (TIMP) in experimental autoimmune encephalitis (EAE) [8]. Given as a rescue therapy, FTY720 reduced BBB leakiness after disease onset [8]. Studies in other vascular beds have shown that FTY720 can protect endothelial cells from apoptotic cell death [9]. However, there are conflicting data on the net effect of S1P receptor agonism on endothelial barrier function, with FTY720 being protective in a model of pulmonary edema caused by systemic LPS injection [10] while it has deleterious effects in a model of bleomycin-induced pulmonary fibrosis [11].

A neuroprotective effect of FTY720 in the acute phase of stroke has been shown in several experimental studies [12-14]. From a translational point of view, every neuroprotectant that is aimed at ameliorating acute brain damage in the first hours within stroke onset should be tested in conjunction with rt-PA. Therefore, the aim of this study was to assess the effect of FTY720 in conjunction with rt-PA treatment in an experimental model of stroke and its effect of stroke- and rt-PA-induced BBB disruption and matrix metalloproteinase expression.

## Methods

### Experimental model of middle cerebral artery occlusion

C57Bl/6 mice (Charles River Laboratories, Sulzfeld, Germany) were used at 10–12 weeks of age. All experiments were approved by the local governmental authorities (Regierungspräsidium Darmstadt, Germany, approval number F143/51) and conducted in accordance with the National Institutes of Health Guide for the Care and Use of Laboratory Animals. Mice were subjected to transient middle cerebral artery occlusion (MCAO) as described previously [15]. Briefly, mice were anesthetized with 1.5–2.5% isoflurane (Forene; Abbott, Wiesbaden, Germany) and 0.1 mg/kg buprenorphine (Temgesic®; Essex Pharma, Munich, Germany) under spontaneous respiration. Focal cerebral ischemia was induced by inserting a custom made filament with a tip diameter of 0.23 mm (Doccol, Sharon, USA) into the middle cerebral artery (MCA). Regional cerebral blood flow was monitored by laser Doppler flowmetry (PF5010, Perimed; Stockholm, Sweden) to confirm vessel occlusion. After an occlusion time of 3 h, the filament was withdrawn to initiate reperfusion. After the operation, mice were allowed to recover from anesthesia with free access to food and water. At the end of a 24 h observation period, mice were lethally anaesthetized and perfused transcardially with PBS. Brains were removed quickly and divided into ischemic and non-ischemic hemispheres before they were frozen and stored at −80°C to await further procedures. Brains from mice that had died within the 24 h observation period were harvested without prior transcardial perfusion.

### Sample size calculation, experimental groups and substance application

We based our sample size calculation on the quantifiable parameters Evans Blue extravasation and matrix metalloproteinase-9 activatity. Assuming an increase of 25% in these two parameters between the ischemic hemispheres of the vehicle-treated and rt-PA-treated mice that has been shown in previous studies and a standard deviation of 33% of the respective mean values (Cohen’s D 0.85), a group size of 18 animals was necessary to show this effect with a power of 0.8 and a probability of a type I error of < 0.5 [16]. After the MCAO operation and prior to reperfusion, we randomized 18 mice per group into the four treatment groups: 1) vehicle-treatment, 2) 1 FTY720 1 mg/kg 3) rt-PA 10 mg/kg, or 4) FTY720 and rt-PA as a combination therapy. The mice received either FTY720 (1 mg/kg, dissolved in 0,9% NaCl; Fingolimod; Cayman Chemicals, Ann Arbor, USA) or saline i.p. in combination with an i.v. bolus of either rt-PA (10 mg/kg; Actilyse®; Boehringer Ingelheim, Ingelheim, Germany) or aqua ad injectabilia. The operator was blinded to the pharmaceutical treatment during the whole study. Additionally, we assessed whether preconditioning of the rt-PA-solution by exposure to a fibrin-containing clot would enhance its deleterious effects on the blood–brain barrier. To this end, blood was drawn from a donor mouse to generate a spontaneously-formed blood clot. Rt-PA was incubated with this blood clot for 30 min under gentle shaking, generating “activated” rt-PA (Art-PA). We performed 3 h of MCAO in two sets of mice that received an i.v. bolus of either rt-PA (10 mg/kg) or Art-PA (10 mg/kg).

### FACS analysis of lymphocyte counts

500 μl of blood were drawn into EDTA-coated tubes from 3 animals per group (1 mg/kg FTY720 vs. saline) 2 h after the injection of FTY720 or vehicle, cells were spun down and erythrocytes were lysed with red cell lysis buffer (8,3 g NH4Cl in 0,01 mol/L Tris–HCl, pH 7,4). After washing with RPMI, samples were incubated with FACS buffer (1% FCS in PBS/0,01% NaN3) and the respective antibodies (Rat anti-mouse CD4 [APC-coupled, Southern Biotech, 1540–11], rat anti-mouse CD8 [PerCP-CTM5.5-coupled, BD Pharmingen, 551162]) for 30 minutes before overnight fixation in 4% PFA in PBS at 4°C. Samples were analyzed on a FACSCalibur with the CellQuestPro Software (BD Biosciences).

### Evaluation of neurological function

Neurological function was evaluated at 3 h directly prior to reperfusion and at 24 h post-MCAO on a 14 point scale modified from Chen et al. [17] testing hemiparesis, gait, coordination and sensory functions (Additional file 1: Table S1).

### Determination of blood–brain barrier leakage

To assess blood–brain barrier leakage, the extravasation of the autofluorescent dye Evans Blue (EB) that binds to plasma albumin was quantified from brain hemispheres as described before [5]. 23 h after MCAO, 200 μl of 2% Evans Blue in 0.9% NaCl were injected into the retrobulbar venous plexus and allowed to circulate for 1 h prior to transcardial perfusion and brain removal. Brain samples were homogenized in lysis buffer as described above and additionally with ultrasound. Protein precipitation was obtained by adding 50% trichloroacetic acid. The supernatant was diluted 4fold with ethanol. The amount of Evans blue dye was measured by a microplate fluorescence reader (excitation 600 nm, emission 650 nm; SpectraMax M5; Molecular Devices, Sunnyvale, USA).

### MMP-9 activity

Gelatin zymography was used to measure the levels of MMP-9 activity in the brain samples as described previously [5,18]. We analyzed the brains of all mice, including those that did not survive the observation period. Brain hemispheres were homogenized in ice-cold lysis buffer contanining protease inhibitors. After centrifugation, the supernatant was collected and total protein concentration of each sample was determined by the Bradford assay (Nanoquant, Roth, Karlsruhe, Germany). Equal volumes of total protein extracts in sample buffer (4% SDS, 0.005% bromphenol blue, and 20% glycerol) were loaded onto 10% polyacrylamide gels containing 0.1% gelatin as a substrate. After electrophoresis, the gels were incubated in 2.5% Triton X-100 at room temperature for 30 min with gentle agitation and stained with 0.5% Coomassie Blue G250. For densitometry, gels were scanned, inverted and integrated density of the bands was quantified with NIH image J 1.44p.

### MMP-9 protein expression

We evaluated MMP-9 expression on the protein level by Western Blotting. Samples of equal total protein content were loaded onto polyacrylamide gels. After migration, proteins were electrotransferred onto a nitrocellulose blotting membrane. To confirm successful transfer, the membrane was stained with Ponceau S. It was then blocked in 5% dry milk and 0.05% Tween-20 for 1 h at room temperature under gentle agitation. After extensive washing, the membrane was incubated with the primary antibody (Anti-MMP-9 rabbit polyclonal; Millipore, Billerica, USA) over night. After exposition to a second antibody (goat anti-rabbit antibody; Bio-Rad, Munich, Germany), the blots were developed with a chemiluminescence reagent on hyperfilm.

### Statistical analysis

Graph Pad Prism 4 (Graph Pad Software Inc., La Jolla, CA, USA) was used for statistical analysis. Results are expressed as means +/− standard deviation. Statistical significance of the differences between groups was evaluated with One-way ANOVA with Bonferroni’s correction for parametric values or the Kruskal-Wallis test with Dunn’s correction for nonparametric values.

## Results

### Intraperitoneal injection of FTY720 leads to a rapid decrease of blood lymphocyte counts in mice

**Figure 1:**
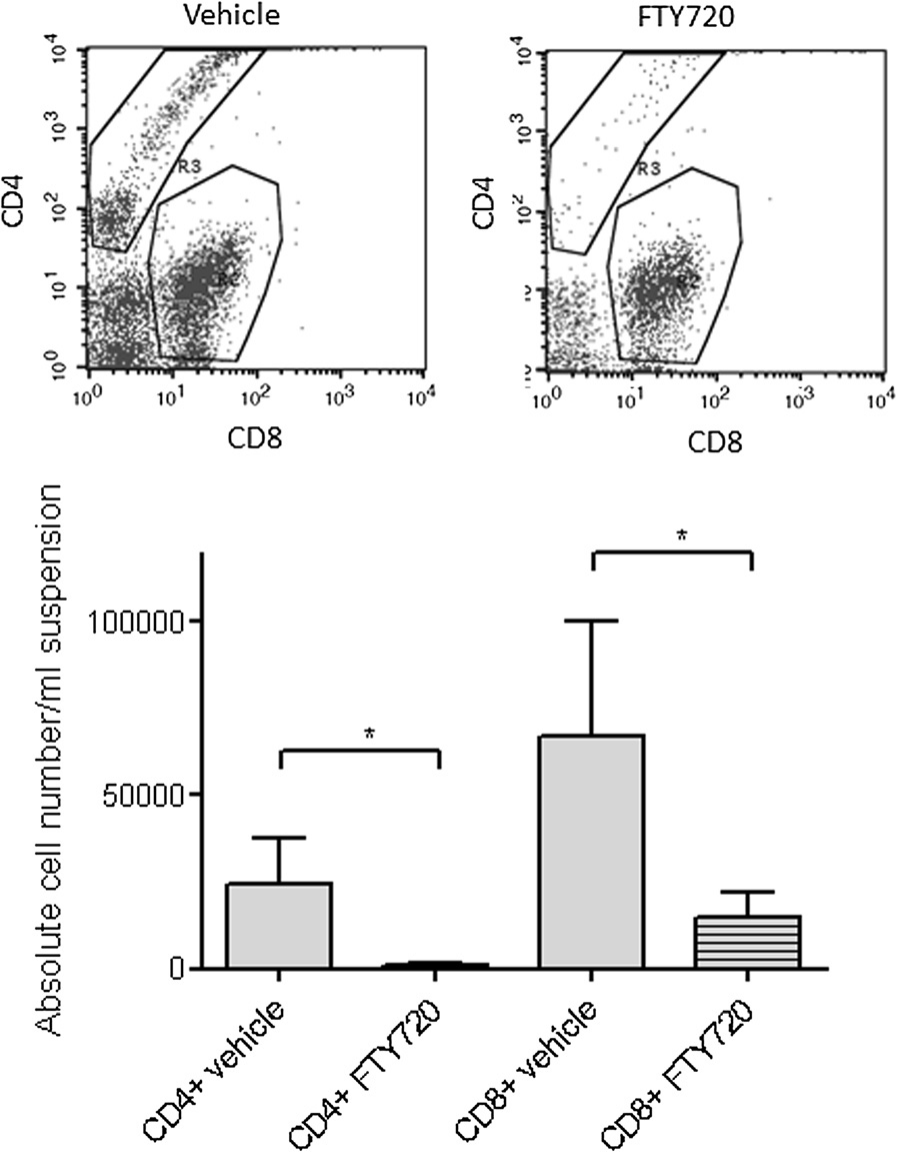
**FTY720 leads to a rapid decrease of blood lymphocyte counts.** We sampled blood 2 h after i.p. injection of FTY720 (1 mg/kg). FACS analysis showed a significant reduction of both CD4+ and to a lesser extent CD8+ T cells (n = 3/group). Statistical significance between groups was tested with two-tailed Student’s *t*-test for unpaired values. * p < 0.05.

To verify efficient uptake and potency of FTY720 via the route of administration that we chose for our experiment, we sampled blood 2 h after i.p. injection of 1 mg/kg FTY720. Fluorescence-activated cell sorting (FACS) analysis of CD4+ and CD8+ T cell populations showed a significant decrease in circulating T lymphocytes to 22.5% for CD8+ T cells and 4.3% for CD4+ T cells as compared to vehicle treated mice (Figure 1).

### FTY720 in conjunction with rt-PA does not improve survival or functional neurological outcome in large hemispheric infarctions

**Figure 2:**
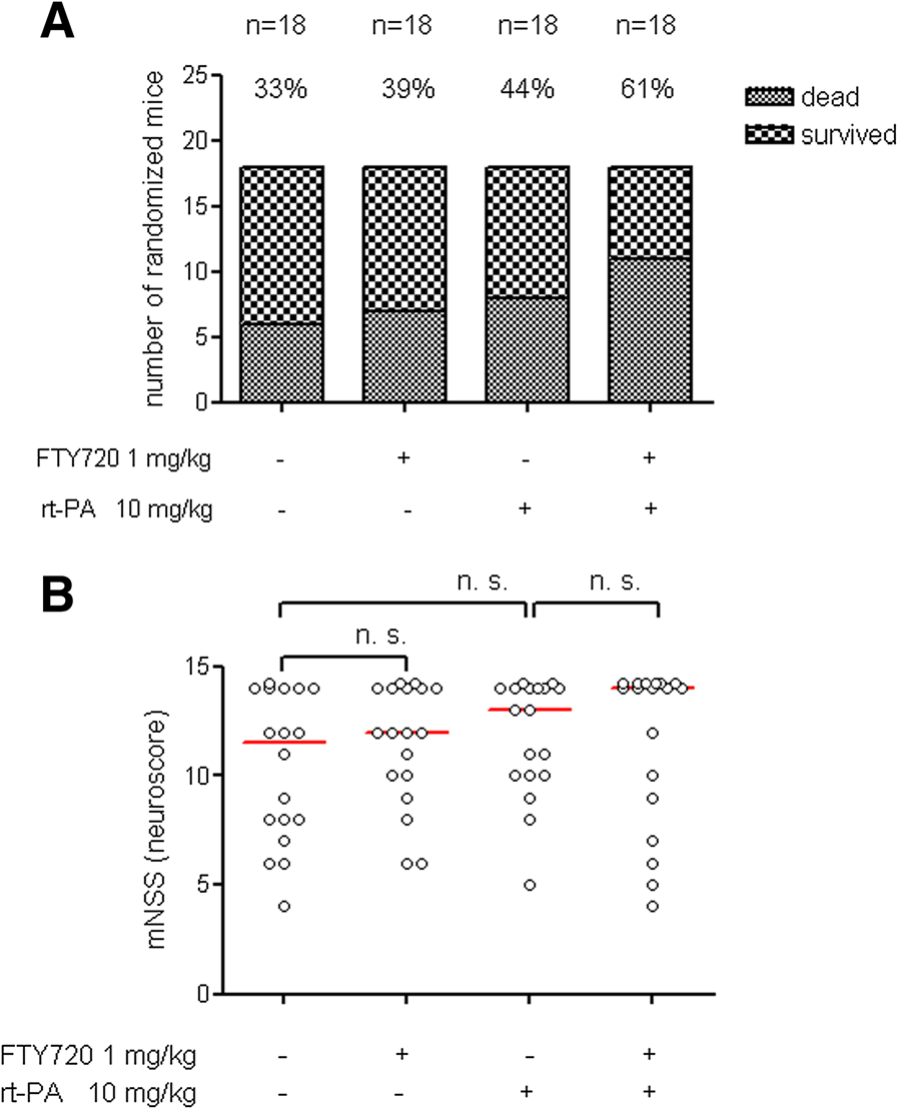
**FTY720 in conjunction with rt-PA does not improve survival or functional neurological outcome in large hemispheric infarctions. A)** Mice were randomized to four different treatment groups (n = 18/group). Operation, treatment and neurological evaluation were performed in a blinded fashion. The mortality rate of each treatment group is given over the respective column. **B)** Functional neurological outcome on a 14-point scoring scale was evaluated at 24 h after the onset of ischemia. Each dot represents an individual mouse. Bars depict median values. Statistical significance of the differences between groups was tested with Kruskal Wallis test with Dunn’s correction for multiple comparisons. ns indicates not significant.

Mice were randomized into four groups (n = 18/group) to receive either 10 mg/kg rt-PA or vehicle i.v. at the end of the 3 h MCAO period in combination with either 1 mg/kg FTY720 or vehicle i.p. The vehicle only-treated group showed a mortality of 33%. Interestingly, the group who received FTY720 in conjunction with rt-PA showed a considerably higher mortality of 61% (Figure 2A). There were no significant differences between groups in the functional neurological examination with the 14-point neuroscore mNSS (Figure 2B).

### FTY720 does not enhance blood–brain barrier integrity in large hemispheric infarcts alone or in combination with rt-PA

**Figure 3:**
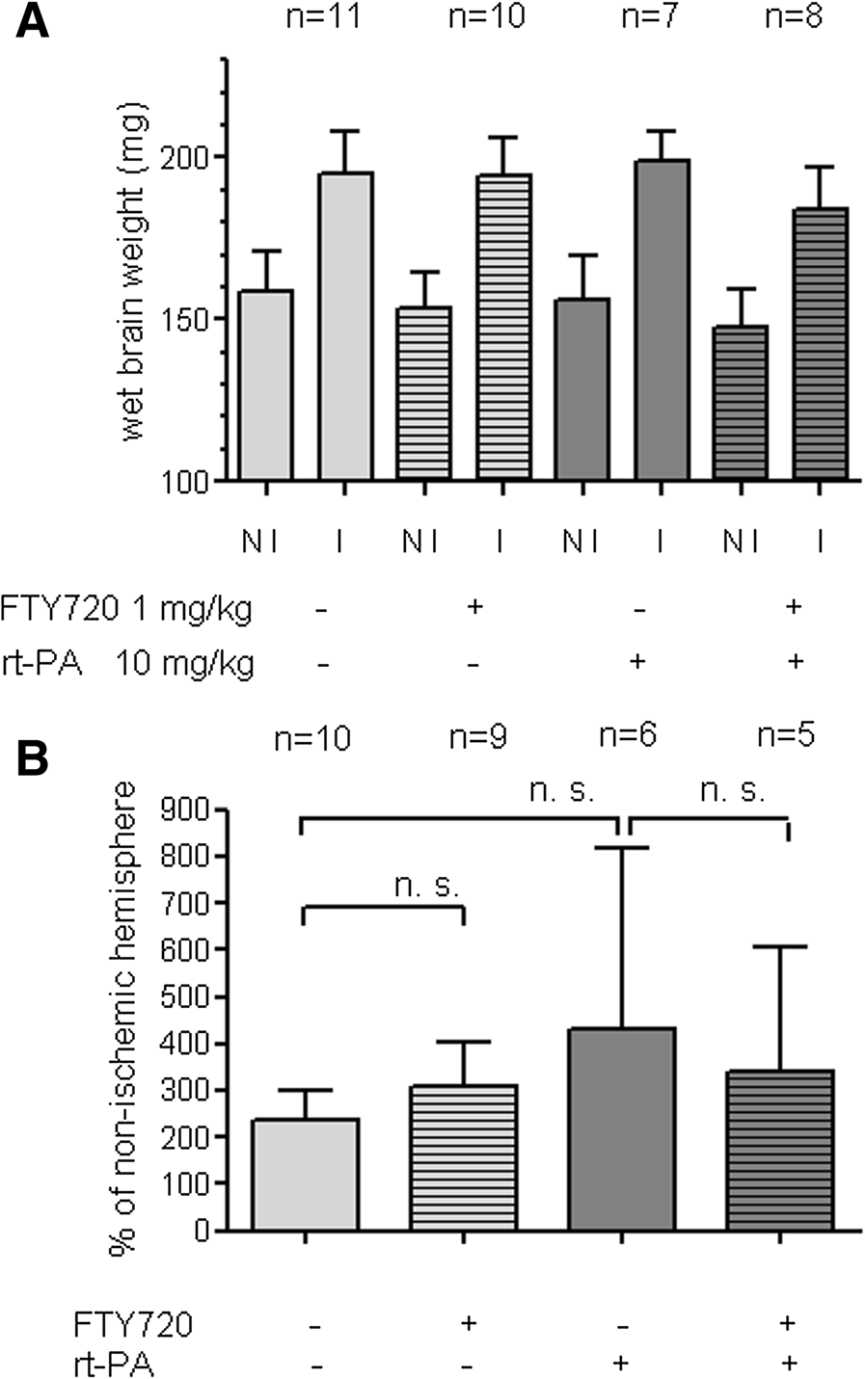
**FTY720 does not enhance blood–brain barrier patency in large hemispheric infarcts neither alone nor in combination with rt-PA. A)** For the determination of wet brain weight, the cerebellum was discarded and brain hemispheres were frozen separately at −80°C. The frozen brain hemispheres were weighed prior to homogenization. Only mice whose ischemic hemisphere (I) showed an increase of wet brain weight of ≥ 10% in comparison to the non-ischemic hemisphere (NI) were included in our analysis. **B)** EB (200 μl of a 2% solution) was injected 1 h prior to sacrifice and transcardial perfusion. We excluded mice who showed more EB extravasation into the NI hemisphere and mice who died within the EB circulation time of 1 h. Statistical significance of the differences between groups was tested with One-way ANOVA with Bonferroni correction. ns indicates not significant.

Brain weight of the ischemic hemisphere was increased by 30–40% in comparison to the non-ischemic hemisphere. Neither rt-PA nor FTY720 treatment alone had an influence on this very crude measure of brain swelling and also rt-PA in combination with FTY720 did not lead to significant changes in wet brain weight. We only included mice whose ischemic hemisphere showed at least a weight increase by 10% to exclude all mice who showed postmortal global brain swelling (Figure 3A) after being found dead during the observation period. All mice who survived the 24 h observation period received 200 μl 2% Evans Blue (EB) i.v. one h prior to sacrifice to assess BBB permeability for macromolecules such as albumin. Fluorometric EB quantification from brain homogenates of transcardially perfused mice did also not show an alteration of stroke-induced EB extravasation by rt-PA, FTY720 or the combination of both substances (Figure 3B).

### Neither rt-PA nor FTY720 induce significant changes in MMP-9 activity in brain homogenates 24 h after MCAO

**Figure 4:**
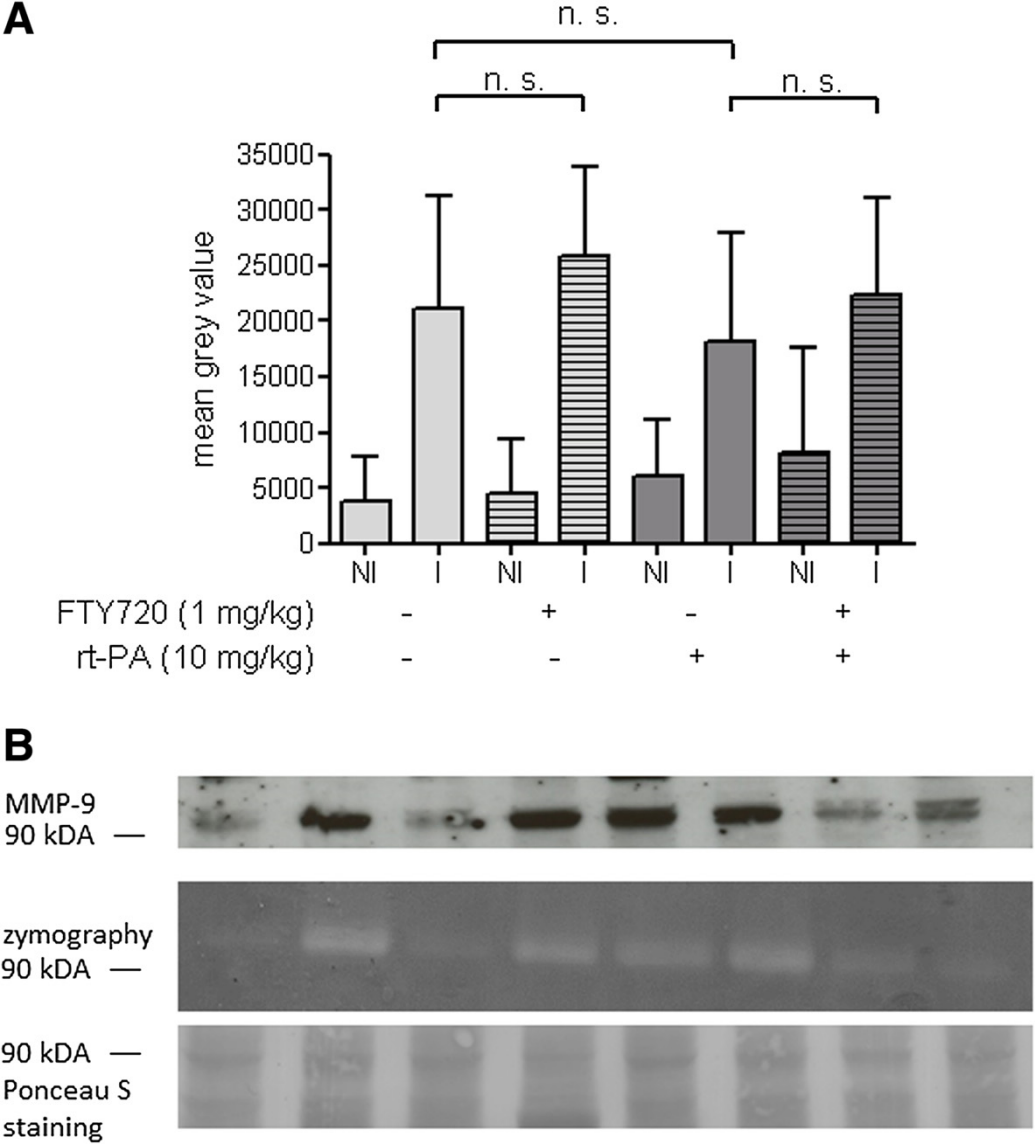
**Neither rt-PA nor FTY720 induce significant changes in MMP-9 activity in brain homogenates 24 h after MCAO. A)** Non-ischemic (NI) and ischemic (I) hemispheres were homogenized separately in ice-cold cell lysis buffer containing protease inhibitors and subjected to acrylamide gel electrophoresis on a gel containing 0.1% gelatin. Photographs of the Coomassie blue-stained gels were inverted and the MMP-9 band at 84 kDa densitometrically evaluated. We excluded all mice who showed greater MMP-9 activity in the NI as compared to the I hemisphere. **B)** We compared MMP-9 expression on the protein level (Western Blot, upper panel) with MMP-9 gelatinase activity (zymography, middle panel) from identical samples of brain tissue. Total protein staining with the azo dye Ponceau S served as a loading control.

Matrix metalloproteinase-9 (MMP-9) is a serine protease that becomes activated in case of ischemic brain injury and has been shown to be a key mediator of blood–brain barrier breakdown in experimental stroke [5]. We performed gelatin zymography to assess MMP-9 activity in the infarcted and non-infarcted hemisphere. While there was a clear, approximately five-fold increase in the infarcted hemisphere, much to our surprise, the administration of FTY720 or rt-PA or the combination of both substances at the end of the 3 h MCAO period did not lead to significant changes in MMP-9 activity (Figure 4A). We performed exemplary Western Blots of MMP-9 and found that MMP-9 activity as assessed by gelatin zymography was highly correlated with MMP-9 protein expression (Figure 4B).

### Activated rt-PA (Art-PA) does not lead to a further increase in blood–brain barrier breakdown after cerebral ischemia

**Figure 5:**
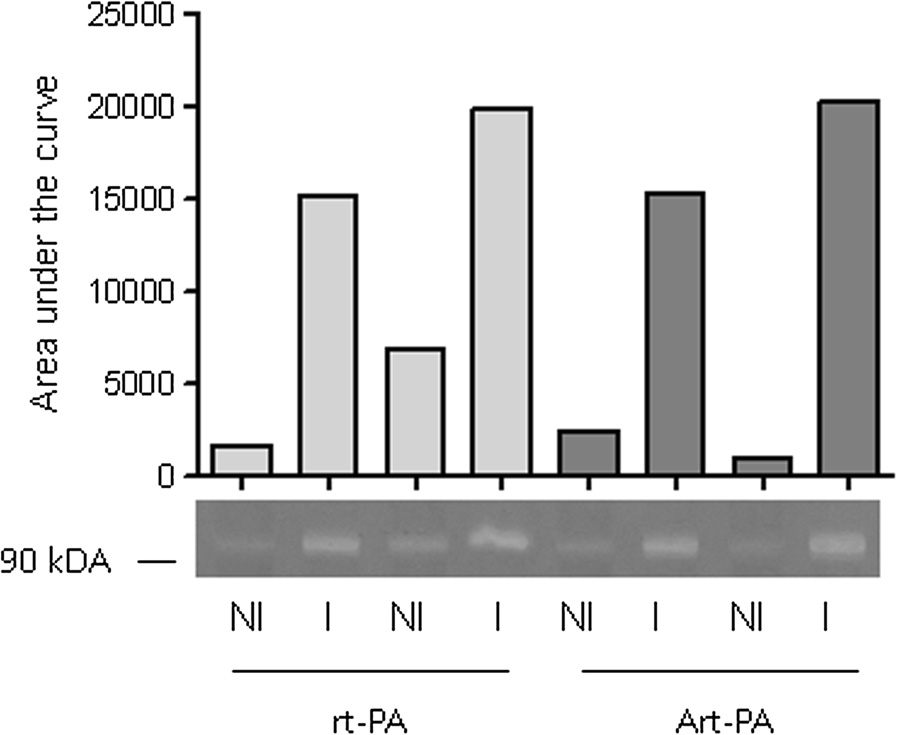
**Activated rt-PA (Art-PA) does not lead to a further increase in blood–brain barrier breakdown after cerebral ischemia.** Rt-PA (1 mg/ml) was either injected directly in the dose of 10 mg/kg in 2 mice after MCAO (first four lanes, homogenates of the non-ischemic and ischemic hemisphere of each mouse) or incubated with a spontaneously-generated blood clot for 30 minutes under gentle shaking prior to injection in 2 mice (last four lanes). MMP-9 activity was assessed with gelatinase zymography. Bands were analyzed densitometrically.

We aimed to clarify the lack of a clear detrimental effect of rt-PA on BBB integrity and MMP-9 activity in our experimental setting. Since it is conceivable that rt-PA only develops its proteolytic effect when it is activated by a fibrin rich clot of adequate surface, we preincubated rt-PA with a spontaneously-formed autologous blood clot for 30 min at room temperature prior to injection after MCAO to generate activated rt-PA (Art-PA). This preconditioning of rt-PA, however, also did not lead to the 2–4fold increase of the cerebral ischemia-induced MMP-9 activity in the ischemic hemisphere that has been reported elsewhere for the filament occlusion model [4] (Figure 5).

## Discussion

We found no relevant protective effect of FTY720 when applied in conjunction with rt-PA in an experimental model of large hemispheric strokes, neither on functional neurological outcome nor on markers of BBB disruption. In contrast, our data rather point towards safety concerns against the coadministration of these two drugs in patients with severe strokes.

Analyzing mortality, we found a noticeable difference between the vehicle group (33%) and the groups with administration of FTY720 alone (39%) or rt-PA alone (44%) on the one hand and the cotreatment group who received rt-PA in conjunction with FTY720 (61%) on the other hand. When evaluating functional outcome after 24 h on the 14 point neuroscore including dead animals which were assigned the maximal score of 14, the differences between groups were not statistically significant.

These data are in some aspects remindful of the results of the multicentric clinical phase II/III trial of eryhtrompoietin (EPO) published in 2009, which assessed its safety and efficacy in acute stroke [19]. This clinical trial was preceded by promising experimental studies that had shown a robust neuroprotective effects and a clinical phase I trial of EPO as a monotherapy in the acute phase of stroke [20] that had demonstrated adequate safety. In contrast, the phase II/III trial, which for the first time allowed systemic thrombolysis in conjunction with EPO treatment, failed to show efficacy. Characteristics of this trial population were rather severe strokes (mean NIHSS: 13) and the coadministration of systemic thrombolysis in 60% of patients [19]. There was an excess of mortality in the group of erythropoietin-treated patients compared to the placebo group and the patients who received erythropoietin as a cotreatment together with thrombolysis fared distinctly worse [19]. Therefore, we interpret our data as a caveat that FTY720, which has shown a beneficial effect on outcome and infarct size in several different stroke models, might not be effective if used as a treatment for severe strokes, especially in conjunction with systemic thrombolysis.

Regrettably, our data do not allow a functional explanation of the excess mortality of rt-PA treatment in conjunction with FTY720. Focusing on BBB analyses, we did not perform a quantification of hemorrhagic transformation, e.g. with brain imaging or a hemoglobin assay. Therefore, we have no information on whether the combination therapy led to an increase in hemorrhagic transformation. Concerning alternative extracranial causes of mortality, FTY720 has been shown to induce bradycardia [21], bronchoconstriction and mild pulmonary edema [22] in mice and humans. Besides that, the paucity of circulating lymphocytes could in principle entrain an increased susceptibility towards infections, even though we showed previously, that FTY720 does not increase the rate of stroke associated pneumonia in mice [23] and similar findings have been shown for equally specific immunomodulatory interventions in experimental stroke [24]. However, these symptoms are no common side effects of rt-PA treatment and should have occurred to the same extent in the group receiving FTY720 alone if they were to explain this excess mortality.

Much to our surprise, we did not detect a deleterious effect of rt-PA alone on stroke induced BBB dysfunction. This is at odds with many experimental studies describing an aggravating effect of rt-PA on BBB disruption after stroke [4,5]. One possible explanation could have been that rt-PA was not biologically active in our experimental system (given as an i.v. bolus injection of 10 mg/kg). We cannot directly prove an effect of rt-PA, but previous studies from our group using the same injection technique showed clear effects of rt-PA on HT after stroke [25]. Interestingly, the increase of BBB disruption after stroke has been shown to be greater in embolic than in mechanical models of stroke as the filament-occlusion used in the present study [4]. There are even reports that the administration of rt-PA in the filament-model *per se* does not increase BBB damage unless it is “activated” by preincubation with a clot [26,27]. However, in our hands, even after preincubation of the rt-PA solution with an autologous blood clot, we did not find a relevant increase in the ischemia-induced MMP-9 activity by activated rt-PA. Therefore, use of preactivated rt-PA did not serve to improve our experimental model.

Against the backdrop of our own observations and the published studies on FTY720 in experimental stroke studies, it is somewhat surprising that we did not find a beneficial effect of FTY720 on functional outcome in this experiment. We ascribe this discrepancy to the long MCAO occlusion time which we chose to induce maximal brain damage in order to sensitively detect HT after rt-PA treatment. We did not measure ischemic lesion size in this study. It is conceivable that while ischemic lesion size was reduced by FTY720, this did not translate into clinical benefit anymore because of the severity of the ischemic insult, explained by a ceiling effect of the functional neuroscore. We ascertained the timely biological efficacy of FTY720 in the chosen mode of application by a quantification of blood lymphocytopenia via FACS analysis, shown in the first figure.

Recently, Campos et al. reported on a beneficial effect of FTY720 in thrombolysis which was only manifest when rt-PA was applied late, i.e. 180 min after vessel occlusion as opposed to 30 min [28]. They made use of distal MCAO by direct thrombin injection that led to circumscript cortical infarctions with reperfusion upon rt-PA treatment. FTY720 alone led to a reduction of lesion size but also the combination therapy of FTY720 and rt-PA applied at 180 min reduced lesion size in comparison to vehicle treatment. Interestingly, they were able to demonstrate that FTY720 reduces rt-PA induced EB extravasation after stroke and rt-PA as a marker for BBB dysfunction, even after normalization for the reduction of lesion size. The main discrepancy between our studies is the size of the infarction produced by the respective experimental model which might have led to a ceiling effect in our case, where a modest protective effect is no longer discernible. We chose large hemispheric infarctions to induce severe BBB damage in order to increase the aggravation of BBB injury by rt-PA.

From a translational point of view, our data point towards the issue that the protective effect of FTY720 in acute stroke may be lost in large hemispheric infarctions. They represent a caveat that the combination therapy of FTY720 and rt-PA might lead to an excess mortality.

## Competing interests

WP receives project-specific funding from Novartis Pharma Nürnberg, Germany for another project not related to the data presented in this manuscript.

## Authors’ contributions

AC participated in designing the study, conducted the experiments and contributed to the manuscript, FS conducted the blinded evaluation of endpoints, FB participated in designing the study and contributed important intellectual content, SK conducted experiments and contributed important intellectual content, CF participated in designing the study and writing the manuscript, WP designed the study, performed statistical evaluation of the results and wrote the manuscript. All authors read and approved the final manuscript.

## Supplementary Material

Additional file 1: Table S1 Neurological Deficit Score. Click here for file
